# FGF19 Regulates Cell Proliferation, Glucose and Bile Acid Metabolism via FGFR4-Dependent and Independent Pathways

**DOI:** 10.1371/journal.pone.0017868

**Published:** 2011-03-18

**Authors:** Ai-Luen Wu, Sally Coulter, Christopher Liddle, Anne Wong, Jeffrey Eastham-Anderson, Dorothy M. French, Andrew S. Peterson, Junichiro Sonoda

**Affiliations:** 1 Department of Molecular Biology, Genentech, Inc., South San Francisco, California, United States of America; 2 Storr Liver Unit, Westmead Millennium Institute, University of Sydney, Sydney, New South Wales, Australia; 3 Department of Assay and Automation Technology, Genentech, Inc., South San Francisco, California, United States of America; 4 Department of Pathology, Genentech, Inc., South San Francisco, California, United States of America; Istituto Dermopatico dell'Immacolata, Italy

## Abstract

Fibroblast growth factor 19 (FGF19) is a hormone-like protein that regulates carbohydrate, lipid and bile acid metabolism. At supra-physiological doses, FGF19 also increases hepatocyte proliferation and induces hepatocellular carcinogenesis in mice. Much of FGF19 activity is attributed to the activation of the liver enriched FGF Receptor 4 (FGFR4), although FGF19 can activate other FGFRs in vitro in the presence of the coreceptor βKlotho (KLB). In this report, we investigate the role of FGFR4 in mediating FGF19 activity by using Fgfr4 deficient mice as well as a variant of FGF19 protein (FGF19v) which is specifically impaired in activating FGFR4. Our results demonstrate that FGFR4 activation mediates the induction of hepatocyte proliferation and the suppression of bile acid biosynthesis by FGF19, but is not essential for FGF19 to improve glucose and lipid metabolism in high fat diet fed mice as well as in leptin-deficient ob/ob mice. Thus, FGF19 acts through multiple receptor pathways to elicit pleiotropic effects in regulating nutrient metabolism and cell proliferation.

## Introduction

FGF19 (fibroblast growth factor 19) and its murine ortholog Fgf15 are the founding members of the endocrine FGF subfamily that also includes FGF21 and FGF23 [1], [2]. FGF19 and Fgf15 influence a variety of metabolic processes including glucose, lipid and bile acid (BA) metabolism as well as gall bladder filling [3], [4], [5], [6]. Transgenic overexpression of FGF19 in mouse skeletal muscle results in the accumulation of FGF19 in serum, and reverses high fat diet (HFD)-induced weight gain and various metabolic defects associated with obesity, including hepatic lipid accumulation, insulin resistance, and increased serum lipid levels [3], [4]. Treatment of leptin deficient ob/ob mice or HFD-induced obese mice with recombinant FGF19 causes an increase in metabolic rate, resulting in weight loss, decreased hepatic triglyceride content and a dramatic improvement in glucose utilization and insulin sensitivity [3]. Very similar metabolic effects have more recently been described for FGF21, the most closely related member of the FGF superfamily to FGF19 (and murine Fgf15), suggesting that FGF19 and FGF21 may act through a common receptor pathway [7], [8], [9].

In addition to the effects on lipid and glucose metabolism, FGF19/Fgf15 has also been implicated in the regulation of hepatic BA metabolism and hepatocyte proliferation. FGF19/Fgf15 expression in the intestine is transcriptionally regulated by the nuclear BA receptor Farnesoid X Receptor (FXR) [5], [10] and is induced in the post-prandial state in humans, when BA levels rise in the lumen of the distal small intestine [11]. Circulating FGF19/Fgf15 in turn induces the hepatic expression of atypical nuclear receptor Small Heterodimeric Partner (SHP) to suppress expression of Cyp7a1 and Cyp8b1 encoding cholesterol 7α-hydroxylase and Sterol 12 alpha-hydroxylase respectively, two key enzymes for hepatic BA synthesis [3], [5], [10], [12], [13]. Accordingly, Fgf15 deficient mice exhibit an increase in fecal BA excretion [5], and conversely, hepatic overexpression of Fgf15 reduces fecal BA excretion [14]. Recombinant FGF19 also has the ability to induce hepatocyte proliferation in the liver as measured by BrdU incorporation into DNA [15], [16], [17]. Indeed, FGF19 expressing transgenic mice exhibit increased hepatic BrdU incorporation and elevated expression of α-fetoprotein (AFP) mRNA, a marker for hepatocyte proliferation, as early as 2 months of age, and go on to spontaneously develop hepatocellular carcinomas [15].

FGF19/Fgf15 is believed to act by activating FGF receptor (FGFR) homodimers complexed with a membrane bound protein βKlotho (KLB) [13], [18], [19]. Humans and mice possess four conserved FGFR genes, of which FGFR1-3, but not FGFR4, are alternatively spliced in the extracellular ligand binding domain to yield two principal isoforms, b and c, each exhibiting distinct ligand binding specificity [2]. In the presence of KLB, FGF19 can activate FGFR1c, 2c, 3c, and 4 [18]. Of these four FGFR isoforms, FGFR4 appears to mediate many, if not all, FGF19 activities. Fgfr4 deficient mice exhibit elevated Cyp7a1 mRNA expression, BA excretion and pool size [20] and fail to suppress Cyp7a1 expression upon injection of adenovirus expressing Fgf15 [5]. Furthermore, our unpublished results show that FGF19 transgenic mice do not develop hepatocellular carcinoma in Fgfr4 deficient background (French DM, in preparation). Whether other FGFRs mediate FGF19 activity in vivo is currently not clear. In this report, we investigate the requirement for FGFR4 in mediating FGF19 activity by using Fgfr4 deficient mice as well as a protein variant of FGF19, which is specifically impaired in its ability to activate FGFR4. Our results indicate that FGFR4 activation is essential for some of the activities of FGF19, but is dispensable for the beneficial effects of FGF19 on glucose and lipid metabolism, demonstrating the existence of an FGFR4-independent pathway of FGF19 action.

## Results

### FGFR4 is required for regulation of serum bile acids, but not for improvement of glucose tolerance, by recombinant FGF19

**Figure 1:**
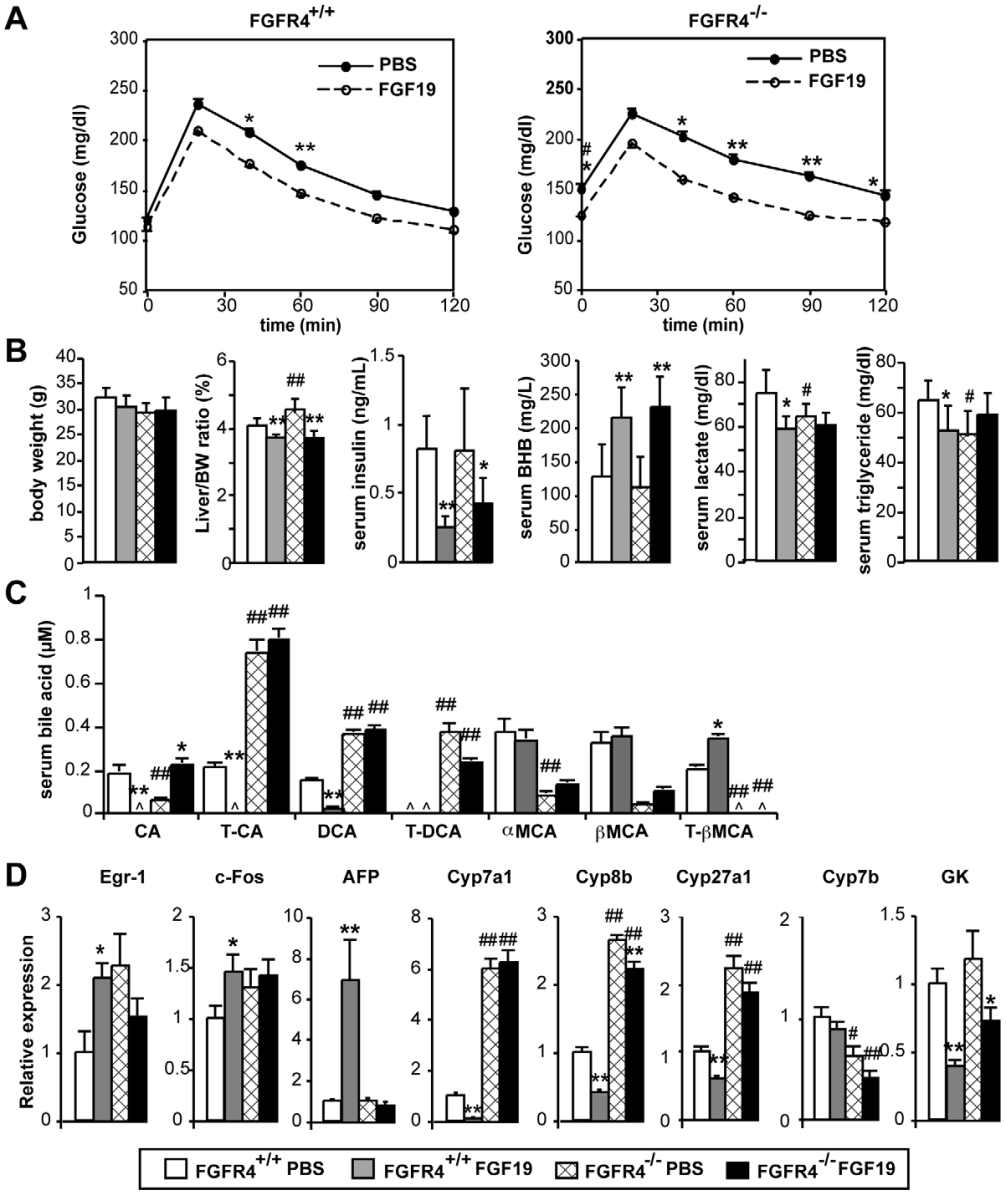
Fgfr4 is required for BA regulation but not for improvement of glucose tolerance by FGF19. (A) 12 to 15 week old Fgfr4 WT and KO mice on high fat diet for 6 weeks were implanted with an osmotic pump to infuse FGF19 at 1 ng/hr (day 0). On day 6, overnight fasted mice were subjected to glucose tolerance test with i.p. injection of glucose at 1 g/kg. *p<0.05. **p<0.01. p value for area under the curve (AUC) was p<0.02 (WT) and p<0.005 (KO). N = 6∼8. Note that fasting blood glucose levels were significantly higher in PBS-treated Fgfr4 KO mice compared with WT mice (#p<0.01). (B) Metabolic parameters at euthanasia on day 7. Mice were euthanized and serum prepared after 3 hr fast. N = 6∼8. (C) Serum BA composition analysis. Only major BA species are shown. CA: cholic acid, DCA: deoxycholic acid, MCA: muricholic acid, T-:taurine-conjugated. ^: undetected. (D) Hepatic gene expression determined by real-time qPCR. N = 6∼8. p values for (B), (C), and (D): <0.05, **<0.005 (PBS vs FGF19), #<0.05, ##<0.005 (WT vs Fgfr4KO).

In order to determine which of the metabolic effects elicited by FGF19 are mediated by FGFR4, we treated HFD-fed WT or Fgfr4 KO mice with recombinant FGF19 or vehicle control and studied metabolic phenotypes and gene expression. To achieve sustained exposure to FGF19, mice were implanted with osmotic pumps to continuously infuse FGF19 at 1 ng/hr. This achieved an average FGF19 serum concentration of 26 ng/ml, as determined by ELISA, about 50- to 250-fold higher than circulating FGF19 concentrations in humans [11]. On day 6, a glucose tolerance test was conducted after overnight fasting. FGF19 infusion improved glucose tolerance to a similar extent both in WT and Fgfr4 KO mice (Fig. 1A), indicating that FGFR4 is dispensable for the improvement in glucose tolerance in HFD-fed mice. Continuous infusion of FGF19 did not induce significant weight loss, thus the improved glucose tolerance was independent of body weight. By day 7, FGF19 reduced liver weight and serum insulin and increased ketone body (β-hydroxybutyrate: BHB) formation in both WT and Fgfr4 KO mice (Fig. 1B). The mock-treated Fgfr4 KO mice exhibited reduced lactate and triglyceride levels compared to WT mice, and FGF19 reduced these parameters in WT but not Fgfr4 KO mice (Fig. 1B).

To evaluate changes in systemic BA regulation, serum BA composition was determined by liquid chromatography-mass spectrometry (Fig. 1C). Although serum BA composition is influenced by a number of factors including hepatic synthesis and transport in the liver, kidney and intestine [21], [22], our finding is consistent with a shift of BA synthesis from the neutral to the alternative (acidic) pathway, bypassing FGF19-suppressed Cyp7a1 and proceeding though Cyp7b1 (Fig. 1D). FGF19 infusion in WT mice reduced free and taurine conjugated cholic acid (CA) and the CA-derived secondary bile acid deoxycholic acid, while having minimal effect on chenodeoxycholic acid (CDCA) metabolites, muricholic acids (MCA). Correspondingly, loss of Fgfr4 increased basal levels of CA and its metabolites while reducing MCA (hydroxylated metabolites of CDCA), indicating that FGFR4 is not only important as a regulator of bile acid synthesis, but is also a determinant of the ratio of CA to CDCA production. To determine the role of FGFR4 in regulation of hepatic gene expression, we examined a range of hepatic mRNAs by qPCR (Fig. 1D). FGF19 infusion induced expression of cell proliferation markers such as Egr-1, c-Fos, and AFP, and suppressed expression of Cyp7a1 in WT but not in Fgfr4 KO mice. In contrast, FGF19 suppressed Cyp8b1 and glucokinase (GK) in both WT and Fgfr4 KO mice, while basal expression of Cyp8b1 and Cyp27a1 levels were much higher in Fgfr4 KO compared to WT mice. The higher basal expression of the Cyp genes is most likely due to loss of suppression by endogenous Fgf15 protein. Although an increase in basal expression was also observed for Egr-1, this was not reproduced in other experiments (see below). Cyp8b1 is obligatory for the synthesis of cholic, but not CDCA [23], thus the observed changes in Cyp8b1 expression contribute to the altered balance between CA and CDCA metabolites (muricholic acids) in Fgfr4 KO mice (Fig. 1C and D). Taken together, our findings reveal that FGFR4 is a pivotal regulator of BA synthesis and impacts hepatocyte proliferation, but is not required for the regulation of glucose utilization, insulin sensitivity, and ketone body production by FGF19.

### Identification of FGF19 variants with a specific reduction in FGFR4 activation

**Figure 2:**
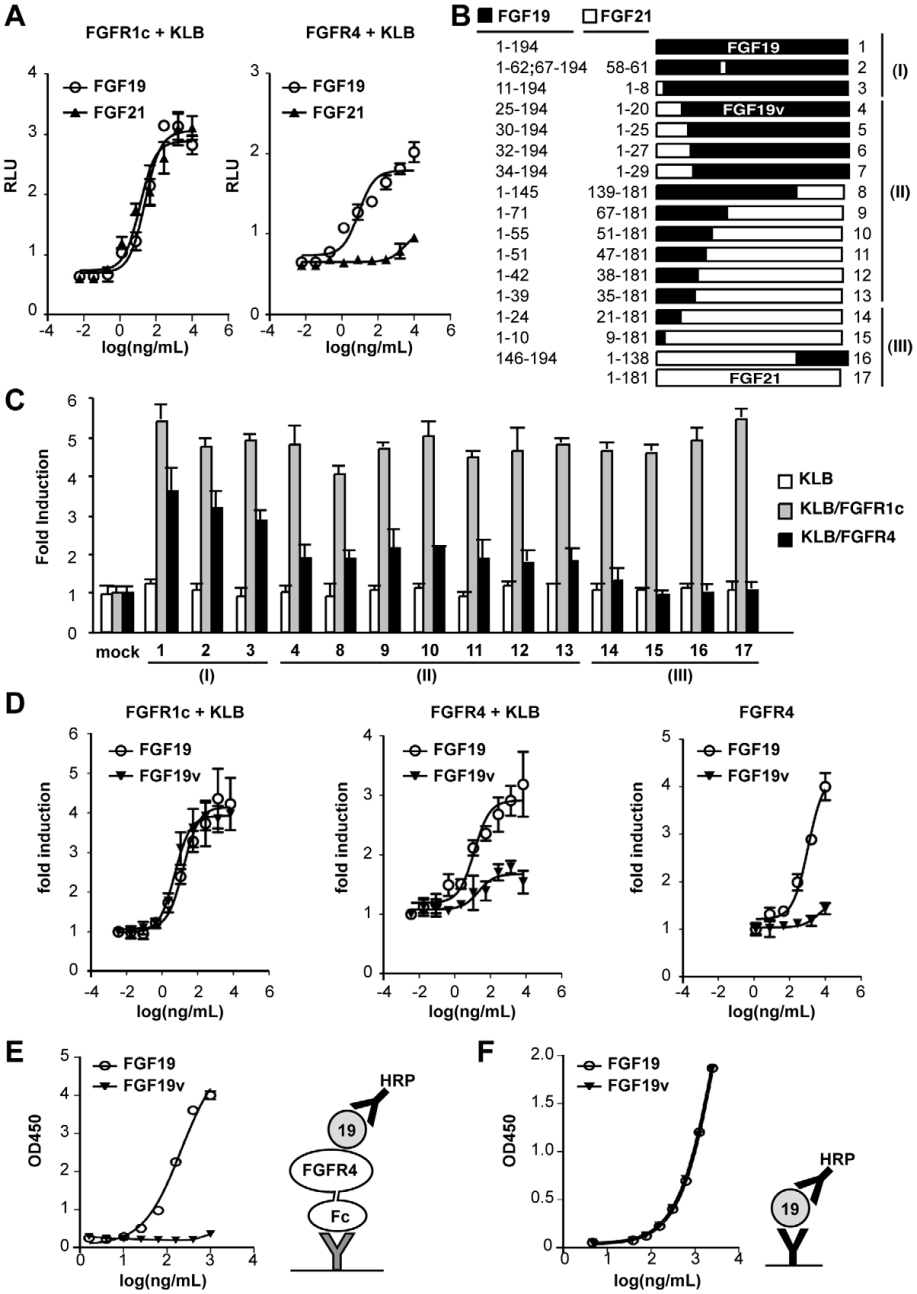
Identification of FGF19 variants with reduced FGFR4 activity. (A) GAL-Elk1 luciferase assay in rat L6 cells. L6 cells were cotransfected with expression vectors for KLB and the indicated FGFR together with GAL-Elk1, SV40-renilla Luciferase, and Gal-responsive firefly luciferase reporter. Transfected cells were incubated with media containing increasing concentrations of FGF19 (○) or FGF21(▴) for 6 hours before luciferase assays. Transcriptional activation was assessed by the relative firefly luciferase activity normalized by renilla luciferase activity and expressed as relative luciferase unit (RLU). (B) Drawings (to scale) of FGF19 (top), FGF21 (bottom), and various chimeric proteins with amino acid composition at left. Based on the results of repeated GAL-Elk1 assays such as shown in (C), each chimera was classified into class (I), (II) or (III) as indicated at right (see text). Chimeras which did not exhibit an equivalent FGFR1c activity to FGF21 or FGF19 when conditioned medium was used were not shown here. (C) Representative results of GAL-Elk-1 assay for chimeras shown in (B). L6 cells were cotransfected with expression vectors for KLB and/or FGFR as indicated at right. Each FGF construct was expressed in transiently transfected 293 cells and the conditioned medium was used in the assay. The results are shown as a fold induction over control media conditioned with mock transfected cells. (D) Similar to (A). Purified FGF19 (○) and FGF19v (▾), (the construct #4 in (B) and (C)), were tested for FGFR activation in the presence or absence of KLB coexpression as indicated. (E) Solid phase binding assay of FGF19 and FGF19v to FGFR4 fused to Fc fragment was tested as described in the method section. Schematic diagram for the experiments is shown at right. Bold Y indicates antibody against FGF19 (black) or Fc fragment (gray). HRP: horseradish peroxidase. (F) A control ELISA experiment to show that anti-FGF19 antibody used in (E) recognize FGF19 and FGF19v at indistinguishable affinity. Schematic diagram for the experiments is shown at right.

Based upon the results described above, we hypothesized that if we could generate FGF19 variants with specifically reduced FGFR4 activity, such molecules would retain beneficial metabolic effects while losing FGFR4-dependent actions such as the induction of hepatocyte proliferation and altered BA homeostasis. In order to quantitatively evaluate specific activation of FGFRs by FGF19, an FGF-responsive GAL-Elk1 luciferase reporter assay was introduced into rat L6 cells [24]. In this assay, effective binding of a ligand to FGFR results in activation of an endogenous MAP kinase pathway, leading to subsequent activation of a chimeric transcriptional activator comprising of an Elk-1 activation domain and a GAL4 DNA-binding domain. L6 cells lack functional FGFR or KLB and are only responsive to FGF19 or FGF21 when cotransfected with cognate receptors [18]. Using this assay, we observed that FGF19 and FGF21 activated FGFR1c, 2c and 3c in the presence of KLB, with similar potency and efficacy (Fig. 2A and S1). In contrast, FGF19, but not FGF21, efficiently activated FGFR4, even in the presence of KLB (Fig. 2A). To map the signals required for FGFR4 activation, we generated a number of chimeric constructs between FGF19 and FGF21 using conserved residues to form junctions (Fig. 2B). Constructs were expressed in HEK293 cells and the culture supernatants containing secreted chimeric FGF proteins were tested for activation of FGFR1c and/or FGFR4 in KLB-expressing L6 cells using the GAL-Elk1 reporter assay. Based on the activity of FGFR1c and FGFR4, the chimeric constructs were classified into 4 classes: high FGFR1c and FGFR4 activity (Class I, FGF19-like); high FGFR1c activity and low, but detectable FGFR4 activity (Class II); high FGFR1c activity without detectable FGFR4 activity (Class III, FGF21-like) (Fig. 2C and S2) and very low or undetectable FGFR1c and FGFR4 activity due to poor expression (Class IV) (not shown). This mapping indicated that the N-terminal 39 amino acids of FGF19 are sufficient to confer some FGFR4 activity when transferred to FGF21. In addition, the N-terminal 24 amino acids and the C-terminal 49 amino acids of FGF19 are necessary for full FGFR4 activity, but are not sufficient to confer FGFR4 activity when transferred to FGF21. Thus multiple signals at both the N-terminus and C-terminus of FGF19 contribute to FGFR4 activation.

One chimeric construct classified as a class II molecule, consisting of amino acids 1-20 of FGF21 and 25-194 of FGF19 (> 90% identical to FGF19), was selected for large scale synthesis in CHO cells and this variant is referred to as “FGF19v”. When compared with FGF19 using the luciferase reporter assay, FGF19v protein exhibited a similar dose-dependent activity to FGF19 in L6 cells cotransfected with KLB and FGFR1c (Fig. 2D). However, FGF19v activity was significantly diminished in L6 cells cotransfected with a combination of FGFR4 and KLB (Fig. 2D). FGF19 activated FGFR4 even in the absence of KLB coexpression in a similar luciferase assay (Fig. 2D), and as previously shown [25], [26], exhibited dose-dependent binding activity to FGFR4 (Fig. 2E and F). However, these activities were largely abrogated for FGF19v (Fig. 2D, E and F).

### FGFR4 mediates hepatocyte proliferation *in vitro* and *in vivo*

**Figure 3 pone-0017868-g003:**
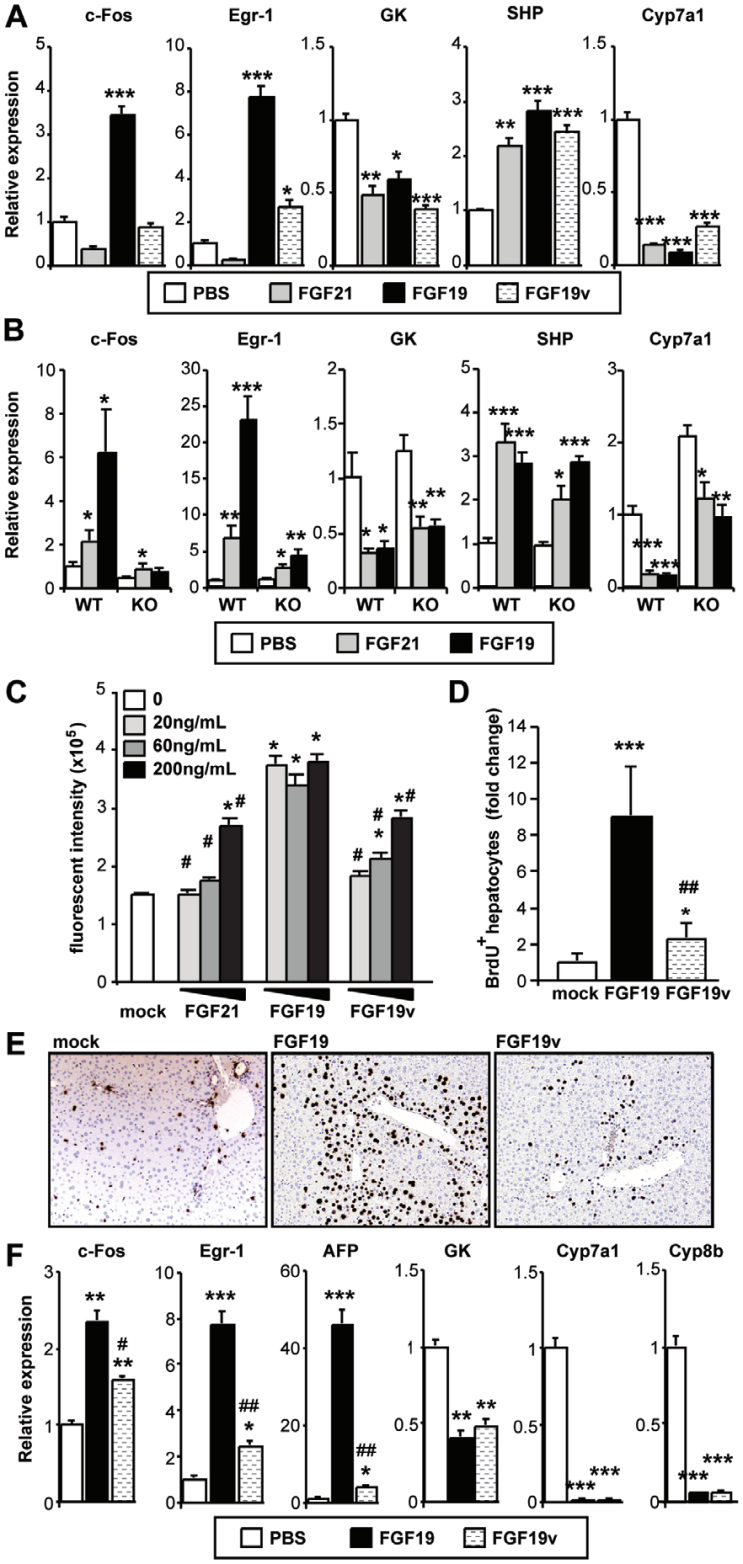
Biological activities of FGF19v in chow-fed lean mice. (A) An acute gene expression study. Overnight fasted FVB mice (N = 5*–*6) were injected via tail vein with indicated FGF protein at 1 mg/kg or PBS control. At 4 hours post-injection, hepatic mRNA was prepared from each mouse and subjected to real-time qPCR analysis for the indicated genes. p values: *<0.05, **<0.01, ***<0.001 (vs PBS) (B) A similar acute gene expression study. Overnight fasted WT or FGFR4 KO mice (N = 5*–*7) were i.p. injected with indicated FGF protein at 1 mg/kg or PBS control. At 4 hours post-injection, hepatic mRNA was prepared from each mouse and subjected to real-time qPCR analysis for the indicated genes. p values: *<0.05, **<0.01, ***<0.001 (vs PBS) (C) Anchorage independent cell growth assay. Proliferation of HepG2 cells in soft agar was estimated based on conversion of Resazurin (Alamer Blue), a non-fluorescent indicator dye, to resorufin. (D) Hepatic BrdU incorporation in FGF treated mice. FVB mice were implanted with an osmotic pump to continuously infuse indicated FGF protein at 1 ng/hr (∼0.8 mg/kg/day) (day 0). The mice also received daily injection of 1 mg/kg/day FGF protein (q.d.) and 30 mg/kg/day BrdU (b.i.d.) starting day 1. On day7, livers were dissected out and subjected to anti-BrdU staining. The results are shown as a fold induction over mock treated animals for the number of BrdU positive hepatocytes per area anlyzed. p values for (C) and (D): N = 6, *p<0.01, ***p<5E−5 (vs PBS), ##p<0.0002 (vs FGF19) (E) Representative images for (C). (F) Hepatic gene expression profile in mice used for (D) and (E). N = 6. *p<0.05, **p<0.005, ***p<0.001 (vs PBS), #p<0.05, ##p<0.005 (FGF19 vs FGF19v).

Activity of FGF19v was further tested in vivo in comparison with FGF19 and FGF21 by intravenously injection into overnight fasted FVB mice. Livers were harvested at 4 hours post injection and hepatic mRNA expression was determined by qPCR. Genes that were acutely induced by FGF19 but not by FGF21, such as Egr-1 and c-Fos, were not efficiently induced by FGF19v, consistent with the reduced FGFR4 activity of FGF19v (Fig. 3A). FGF19v had similar activity to FGF19 or FGF21 on genes co-regulated by FGF19 and FGF21, such as GK. Using Fgfr4 KO mice, we confirmed that FGFR4 contributes to the regulation of Egr-1 and c-Fos, but not GK, by FGF19 (Fig. 3B). Unexpectedly, FGF21 (as well as FGF19 and FGF19v) altered expression of SHP and Cyp7a1 (Fig. 3A), which were proposed to be major targets for FGFR4-dependent regulation by FGF19 [5]. Alterations in SHP and Cyp7a1 by FGF19 and FGF21 were observed even in Fgfr4 KO mice, indicating that with this acute treatment, both endocrine FGFs can modulate expression of these genes through an FGFR4-independent pathway (Fig. 3B).

It has been previously proposed that FGFR4 mediates the induction of hepatocyte proliferation by FGF19 [16] (French, D.M., in preparation). Consistent with this concept, FGF19 increased anchorage-independent proliferation of HepG2 cells in soft agar, and this effect was much less apparent for FGF19v or FGF21 proteins (Fig. 3C). To see whether FGF19v also exhibited reduced ability to induce hepatocyte proliferation in vivo, mice were infused with FGF19, FGF19v (1 ng/h) or vehicle control by osmotic mini-pump. In addition, 1 mg/kg/day of FGF protein was injected intraperitoneally daily for 7 days to the same mice to achieve high peak exposures. To capture intermittent proliferative events, BrdU solution (30 mg/kg) was injected twice daily for a total of 13 injections. Hepatocyte proliferation was determined by measuring BrdU positive hepatocytes in liver harvested on day 7. As previously reported, FGF19 treatment resulted in a dramatic increase in BrdU incorporation; however, this response was significantly blunted for FGF19v (Fig. 3D and E). Hepatic mRNA for Egr-1, c-Fos, and the hepatocyte proliferation marker AFP were all dramatically induced by FGF19 and these inductions were largely absent for FGF19v, while regulation of GK, Cyp7a1 and Cyp8b1 did not differ between FGF19 and FGF19v (Fig. 3F).

### FGFR4 is not required for amelioration of hyperglycemia in ob/ob mice by FGF19

**Figure 4:**
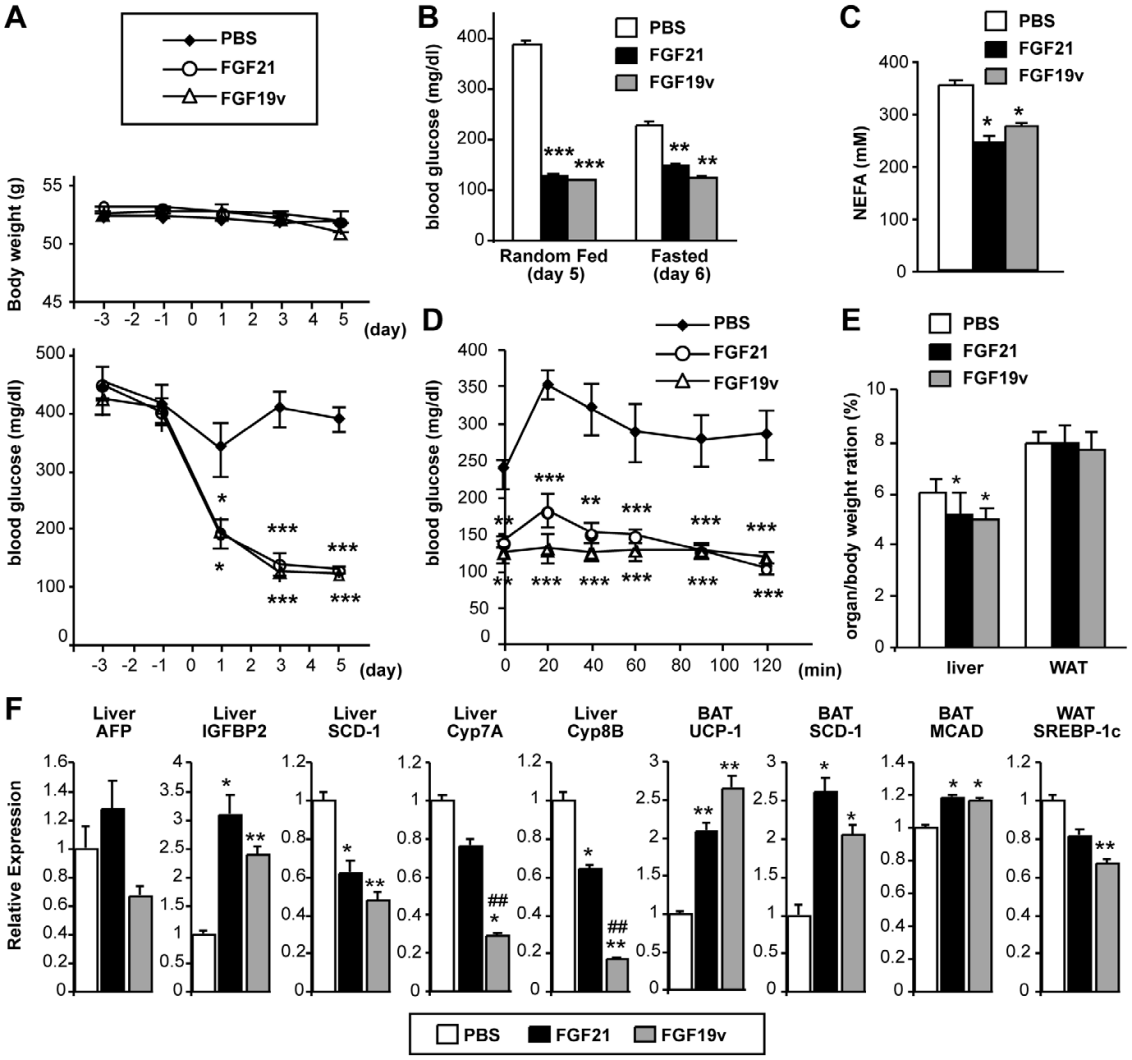
FGF19v and FGF21 exhibit similar metabolic effects and ameliorate hyperglycemia in ob/ob mice. 11-week-old ob/ob mice were subcutaneously implanted with an osmotic pump to infuse 1 ng/hr FGF protein (0.4 mg/kg/day) or PBS control (N = 7). (A) Changes in body weight and random fed blood glucose level. The osmotic pump was implanted on day 0. (B) Blood glucose levels at random fed condition and after overnight fast. (C) Serum non-esterified fatty acids (NEFA) levels on day 8. (D) Glucose tolerance test conducted on day 6. Mice were overnight fasted and i.p. injected with 1 g/kg glucose at t = 0. (E) Organ/body weight ratio on day 8. (F) qPCR gene expression profiles on indicated organs. p values: *<0.05, **<0.005, ***<0.0005 (vs PBS control), ##p<0.005 (FGF21 vs FGF19v).

The in vitro and in vivo results described above raised the question as to whether FGF19v, a variant of FGF19 with reduced FGFR4 activity and proliferative potential, could improve hyperglycemia in diabetic animals similar to FGF21. FGF21, FGF19v (1ng/hr) or vehicle control was continuously infused subcutaneously into ob/ob mice using osmotic mini-pumps. While infusion did not significantly affect body weight (Fig. 4A), both FGF21 and FGF19v dramatically reduced blood glucose levels in both random fed and fasted mice (Fig. 4A and B), reduced circulating free fatty acid levels (Fig. 4C), and improved glucose tolerance (Fig. 4D). In addition, gross liver weight was significantly reduced for both FGF21 and FGF19v treated mice (Fig. 4E). Presumably reflecting a low level FGFR4 activation by FGF19v that we observed in vitro (Fig. 2) and in vivo in lean FVB mice (Fig. 3), Cyp7a1 gene expression was modestly, but significantly suppressed by FGF19v, but not by FGF21 (Fig. 4F). However, no significant change in hepatic expression of AFP mRNA was observed in either FGF-treated groups (Fig. 4F).

The mechanism by which FGF21 and FGF19 ameliorate hyperglycemia in diabetic animals is not well understood. Since FGF21 and FGF19v show very similar anti-diabetic effects in ob/ob mice, we hypothesize that commonly regulated pathways may contribute to their anti-diabetic effects. We identified a number of genes exhibiting commonly altered expression in ob/ob mice treated with FGF21 and FGF19v. In the liver, both proteins induced IGFBP2 (a recently demonstrated anti-diabetic protein) [27], and suppressed stearoyl-Coenzyme A desaturase 1 (SCD-1; a lipogenic gene) and Cyp8b1 (the determinant of the balance between CA and CDCA production) [23]. In addition, they both induced UCP-1 (adaptive thermogenesis), SCD-1 and Medium-Chain Acyl-CoA Dehydrogenase (MCAD; mitochondrial fatty acid oxidation) in brown adipose tissue, and SREBP-1c (lipogenic transcription factor) in white adipose tissue (Fig. 4F). Thus, actions in multiple tissues could mediate the anti-diabetic effects of FGF21 and FGF19v acting through a FGFR4 independent mechanism.

## Discussion

**Figure 5:**
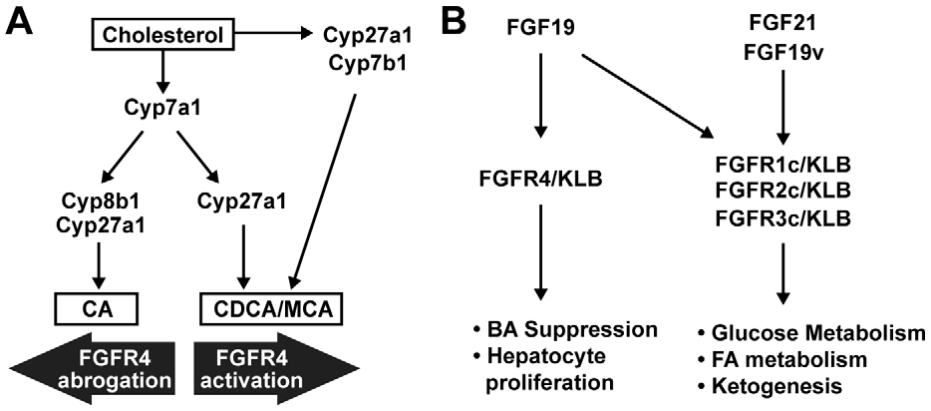
Models for metabolic pathways regulated by FGF19, FGF19v and FGF21. (A) Schematic diagram showing hepatic biosynthetic pathways that convert cholesterol into bile acids. The classical (neutral) pathway (center) is initiated by Cyp7A1, whereas the alternate (acidic) pathway (right) is initiated by Cyp27a1 and Cyp7b1. According to our model, FGF19 suppresses the classical pathway through transcriptional regulation of Cyp7a1 and Cyp8b1, shifting BA synthesis towards production of CDCA or its derivatives. (B) Distinct FGFR/KLB receptor complexes mediate various biological activities of FGF19, FGF19v, and FGF21. In addition to the model depicted, FGF21 and FGF19 can also suppress Cyp7a1 expression acutely in FGFR4-independent manner (Fig. 3).

Although FGF19 has been shown to activate multiple FGFRs in the presence of the coreceptor KLB in vitro, contribution of each FGFR to the in vivo activity of FGF19 has been poorly defined. Our findings in Fgfr4 KO mice as well as using a FGF19 variant protein with reduced FGFR4 activity have delineated pathways downstream of FGF19. We have shown that FGFR4 is required for regulation of BA biosynthesis and hepatocyte proliferation as previously proposed [5], [16]. However, an important additional finding of this work is that FGF19 is also a key qualitative regulator of systemic BA composition. By examining individual serum BA we have demonstrated that recombinant FGF19, acting through Fgfr4, suppresses Cyp7a1 causing bile acid synthesis to proceed by the Cyp7a1-independant alternate (acidic) pathway (Fig. 5A), leading to the production of CDCA at the expense of CA. We also found that Cyp8b1 expression increased several-fold in Fgfr4 knockout mice and that FGF19 treatment suppresses Cyp8b1, an obligatory enzymatic step for CA synthesis (Fig. 5A). It is therefore apparent that FGFR4 is important in determining the ratio of CDCA to CA production, through the negative regulation of both Cyp7a1 and Cyp8b1. FGFR4 activation shifts BA production towards CDCA, while its abrogation leads to CA formation. In addition, FGF19 increased hepatic AFP expression in a Fgfr4 dependent manner, consistent with previous reports [16].

Intriguingly, we found that both FGF19 and FGF21 acutely reduce hepatic expression of Cyp7a1 even in Fgfr4 KO mice (Fig. 3A). This Fgfr4-independent repression is transient, as continuous infusion of FGF19 repressed Cyp7a1 expression only in WT, but not in Fgfr4 KO mice (Fig. 1D), and continuous infusion of FGF21 did not repress Cyp7a1 expression in HFD-fed mice [7] or in ob/ob mice (Fig. 4F). In contrast, Fgfr4-independent repression of Cyp8b mRNA expression by FGF19 or FGF21 was consistently observed even in chronic conditions [7] (Fig. 1D and 4F). One candidate FGFR that might mediate the Fgfr4-independent regulation of Cyp7a and Cyp8b is FGFR2c, which is expressed in the liver [28] and can interact with KLB to form a receptor for both FGF19 and FGF21 [18]. Further work is required to elucidate the biological significance of this Fgfr4-independent Cyp regulation.

Fgfr4 has also been implicated in the regulation of lipid metabolism and glucose tolerance and may indeed mediate regulation of fat metabolism by endogenously produced Fgf15 protein [29]. However, our results indicate that Fgfr4 is unlikely to be important for the regulation of glucose and lipid metabolism by FGF19 at a pharmacological dose. FGF19 improves glucose tolerance in HFD-fed Fgfr4 KO mice (Fig. 1) and FGF19v, a protein specifically impaired of FGFR4 binding and activation, ameliorates hyperglycemia in ob/ob mice (Fig. 2-4). This work compliments previous studies using a FGF19 variant that selectively activates FGFR4 and induces hepatocyte proliferation to suggest the role of FGFR4 for induction of hepatocyte proliferation but not for improvement in glucose metabolism by FGF19 [16], [30]. The activity of FGF19 to improve insulin resistance and hyperglycemia in obese and diabetic mice is shared by a related endocrine FGF, FGF21 [7], [8], [9]. In addition to the effects in insulin resistance and glucose metabolism, FGF19 increases serum BHB levels even in Fgfr4 KO mice (Fig. 1), like FGF21 [31]. Both FGF19 and FGF21 can bind and activate FGFR1c, FGFR2c, and FGFR3c in the presence of KLB [30], [32], [33]. Thus FGFR1c, FGFR2c, or FGFR3c, in cooperation with KLB, are likely to mediate the common metabolic effects of FGF19 and FGF21 (Fig. 5B).

Previously, therapeutic potential for FGF19 in the treatment of obesity and diabetes has been proposed [3], [4]; however, its promotion of hepatocyte proliferation and carcinogenic potential challenges the development of FGF19 for chronic use [15]. With the identification of FGFR4 as the receptor mediating the induction of hepatocyte proliferation but not the improvement in glucose tolerance, it was predicted that FGF19-like molecules with reduced FGFR4 activity should provide metabolic benefits without causing unwanted side effects. Indeed, we have identified a chimeric FGF19v protein that fits these criteria; it ameliorates hyperglycemia and hyperinsulinemia without a detectable increase in hepatic AFP expression in ob/ob mice. When lean and tumor-prone FVB mice were challenged with a supra-therapeutic dose by combination of continuous infusion and daily injection, FGF19v induced only a two-fold increase in hepatic BrdU incorporation compared with vehicle-treated mice, whereas FGF19 induced on average >9 fold increase. While it is not clear how significant this residual level of FGFR4 activity of FGF19v molecule would be in the therapeutic setting, in particular considering humans have endogenous FGF19 protein, further fine mapping of amino acid residues that are important for FGFR4 interaction should help to identify FGF19 variants without residual FGFR4 activity, thus with no proliferative actions. Such proteins would have therapeutic potential for the treatment of insulin resistance, type 2 diabetes, and the broader metabolic syndrome [8].

Each FGF family protein consists of the structurally conserved central globular domain, and the flanking N-terminal and C-terminal segments that are structurally flexible and are divergent in primary sequence. In X-ray crystal structures of multiple FGF/FGFR complexes, the N-terminal segment of the FGF molecule makes specific contact with the FGFR and is believed to play an important role determining the specificity of the FGF-FGFR interaction [34]. Through our efforts to identify specific regions within FGF19 that are important for FGFR4 activation, we found that changing the entire N-terminal segment (amino acid 1–24) of FGF19 to that of FGF21 substantially removes FGFR4 activations without impairing its ability to activate FGFR1 (in the presence of KLB) (Fig. 2). Conversely, changing N-terminal 34 amino acid of FGF21 to the corresponding sequence of FGF19 confers activation of FGFR4. Thus, determinants of receptor specificity reside within the flexible N-terminal segments of FGF19, although other regions within FGF19 are essential for maximum activation of FGFR4. While this manuscript was in preparation, Wu et al. reported an identification of a FGF19 variant with a dramatically reduced ability to activate FGFR4, but that was only modestly compromised for FGFR1c activation and retained the ability to acutely reduce blood glucose levels in ob/ob mice [17]. In this variant called FGF19-4, five amino acids in the N-terminal segment (amino acid 16–20) and 8 amino acids at the N-terminal end of the globular domain (amino acid 28–35) were replaced with the corresponding residues in FGF21, supporting the importance of the N-terminal segments in determination of FGFR-binding specificity. Our systematic and quantitative approach provides further insight to the determinants of functional specificity in the FGF19/FGFR interaction.

In conclusion, our study demonstrates that FGFR4 is not required for beneficial pharmacological activity of FGF19, and that an engineered FGF19 variant mimicking the specificity of FGF21 could successfully be generated. Given the pleiotropic activities of FGF19 and FGF21 on multiple receptors (i.e., FGFR1c, 2c, and 3c), further exploration into altering receptor specificity of FGF19 or FGF21 to achieve specific activation of a particular FGFR may provide a safer and more predictable approach to exploit endocrine FGF pathways and provide new therapeutic options for the epidemic of obesity associated-disorders such as type 2 diabetes, nonalcoholic fatty liver disease and other manifestations of insulin resistance and the metabolic syndrome.

## Materials and Methods

### Research ethics

The study protocols for all animal experiments were approved by the Genentech Institutional Animal Care and Use Committee (IACUC). The approval IDs for this study are: #08-1943, #08-2004, #08-2004A, #08-2004B, #08-2004C, #08-2136, #08-2136F, #09-1001, #09-1066, #10-1818.

### Expression of recombinant FGF protein

Unless otherwise noted, recombinant human FGF21, FGF19 and variants produced in transiently transfected CHO cell and purified to homogeneity in PBS were used for experiments. For some experiments, E. coli derived FGF21 (2539-FG/CF, R&D systems) was used. All the purified proteins were tested for activity by cell based GAL-Elk1 assays prior to use in for other assays. For experiments in Fig. 2B, 2C, and S2, FGF proteins were expressed in transiently transfected HEK293 cells and fresh conditioned serum-free medium was used for assays without purification.

### Amino acid sequences of FGF19, FGF21 and chimeras

All the constructs also possessed a signal sequence at the N-terminal end (cleaved upon secretion) and the flag tag (DYKDDDDK) at the C-terminal end. The sequences derived from FGF21 are shown in bold.

1 (hFGF19): RPLAFSDAGPHVHYGWGDPIRLRHLYTSGPHGLSSCFLRIRADGVVDCARGQSAHSLLEIKAVALRTVAIKGVHSVRYLCMGADGKMQGLLQYSEEDCAFEEEIRPDGYNVYRSEKHRLPVSLSSAKQRQLYKNRGFLPLSHFLPMLPMVPEEPEDLRGHLESDMFSSPLETDSMDPFGLVTGLEAVRSPSFEK

2: RPLAFSDAGPHVHYGWGDPIRLRHLYTSGPHGLSSCFLRIRADGVVDCARGQSAHSLLEIKA**LKPG**TVAIKGVHSVRYLCMGADGKMQGLLQYSEEDCAFEEEIRPDGYNVYRSEKHRLPVSLSSAKQRQLYKNRGFLPLSHFLPMLPMVPEEPEDLRGHLESDMFSSPLETDSMDPFGLVTGLEAVRSPSFEK

3: **HPIPDSS**PHVHYGWGDPIRLRHLYTSGPHGLSSCFLRIRADGVVDCARGQSAHSLLEIKAVALRTVAIKGVHSVRYLCMGADGKMQGLLQYSEEDCAFEEEIRPDGYNVYRSEKHRLPVSLSSAKQRQLYKNRGFLPLSHFLPMLPMVPEEPEDLRGHLESDMFSSPLETDSMDPFGLVTGLEAVRSPSFEK

4 (hFGF19v): **HPIPDSSPLLQFGGQVRQRY**LYTSGPHGLSSCFLRIRADGVVDCARGQSAHSLLEIKAVALRTVAIKGVHSVRYLCMGADGKMQGLLQYSEEDCAFEEEIRPDGYNVYRSEKHRLPVSLSSAKQRQLYKNRGFLPLSHFLPMLPMVPEEPEDLRGHLESDMFSSPLETDSMDPFGLVTGLEAVRSPSFEK

5: **HPIPDSSPLLQFGGQVRQRYLYTDD**PHGLSSCFLRIRADGVVDCARGQSAHSLLEIKAVALRTVAIKGVHSVRYLCMGADGKMQGLLQYSEEDCAFEEEIRPDGYNVYRSEKHRLPVSLSSAKQRQLYKNRGFLPLSHFLPMLPMVPEEPEDLRGHLESDMFSSPLETDSMDPFGLVTGLEAVRSPSFEK

6: **HPIPDSSPLLQFGGQVRQRYLYTDDAQ**LSSCFLRIRADGVVDCARGQSAHSLLEIKAVALRTVAIKGVHSVRYLCMGADGKMQGLLQYSEEDCAFEEEIRPDGYNVYRSEKHRLPVSLSSAKQRQLYKNRGFLPLSHFLPMLPMVPEEPEDLRGHLESDMFSSPLETDSMDPFGLVTGLEAVRSPSFEK

7: **HPIPDSSPLLQFGGQVRQRYLYTDDAQQT**SCFLRIRADGVVDCARGQSAHSLLEIKAVALRTVAIKGVHSVRYLCMGADGKMQGLLQYSEEDCAFEEEIRPDGYNVYRSEKHRLPVSLSSAKQRQLYKNRGFLPLSHFLPMLPMVPEEPEDLRGHLESDMFSSPLETDSMDPFGLVTGLEAVRSPSFEK

8: RPLAFSDAGPHVHYGWGDPIRLRHLYTSGPHGLSSCFLRIRADGVVDCARGQSAHSLLEIKAVALRTVAIKGVHSVRYLCMGADGKMQGLLQYSEEDCAFEEEIRPDGYNVYRSEKHRLPVSLSSAKQRQLYKNRGFLPLSHFLP**LPGLPPALPEPPGILAPQPPDVGSSDPLSMVGPSQGRSPSYAS**

9: RPLAFSDAGPHVHYGWGDPIRLRHLYTSGPHGLSSCFLRIRADGVVDCARGQSAHSLLEIKAVALRTVAIKGV**KTSRFLCQRPDGALYGSLHFDPEACSFRELLLEDGYNVYQSEAHGLPLHLPGNKSPHRDPAPRGPARFLPLPGLPPALPEPPGILAPQPPDVGSSDPLSMVGPSQGRSPSYAS**

10: RPLAFSDAGPHVHYGWGDPIRLRHLYTSGPHGLSSCFLRIRADGVVDCARGQSAHSLL**QLKALKPGVIQILGVKTSRFLCQRPDGALYGSLHFDPEACSFRELLLEDGYNVYQSEAHGLPLHLPGNKSPHRDPAPRGPARFLPLPGLPPALPEPPGILAPQPPDVGSSDPLSMVGPSQGRSPSYAS**

11: RPLAFSDAGPHVHYGWGDPIRLRHLYTSGPHGLSSCFLRIRADGVVDCARGQS**PESLLQLKALKPGVIQILGVKTSRFLCQRPDGALYGSLHFDPEACSFRELLLEDGYNVYQSEAHGLPLHLPGNKSPHRDPAPRGPARFLPLPGLPPALPEPPGILAPQPPDVGSSDPLSMVGPSQGRSPSYAS**

12: RPLAFSDAGPHVHYGWGDPIRLRHLYTSGPHGLSSCFLRIRADG**TVGGAADQSPESLLQLKALKPGVIQILGVKTSRFLCQRPDGALYGSLHFDPEACSFRELLLEDGYNVYQSEAHGLPLHLPGNKSPHRDPAPRGPARFLPLPGLPPALPEPPGILAPQPPDVGSSDPLSMVGPSQGRSPSYAS**

13: RPLAFSDAGPHVHYGWGDPIRLRHLYTSGPHGLSSCFLRIR**EDGTVGGAADQSPESLLQLKALKPGVIQILGVKTSRFLCQRPDGALYGSLHFDPEACSFRELLLEDGYNVYQSEAHGLPLHLPGNKSPHRDPAPRGPARFLPLPGLPPALPEPPGILAPQPPDVGSSDPLSMVGPSQGRSPSYAS**

14: RPLAFSDAGPHVHYGWGDPIRLRHLYT**DDAQQTEAHLEIREDGTVGGAADQSPESLLQLKALKPGVIQILGVKTSRFLCQRPDGALYGSLHFDPEACSFRELLLEDGYNVYQSEAHGLPLHLPGNKSPHRDPAPRGPARFLPLPGLPPALPEPPGILAPQPPDVGSSDPLSMVGPSQGRSPSYAS**

15: RPLAFSDAGP**LLQFGGQVRQRYLYTDDAQQTEAHLEIREDGTVGGAADQSPESLLQLKALKPGVIQILGVKTSRFLCQRPDGALYGSLHFDPEACSFRELLLEDGYNVYQSEAHGLPLHLPGNKSPHRDPAPRGPARFLPLPGLPPALPEPPGILAPQPPDVGSSDPLSMVGPSQGRSPSYAS**

16: **HPIPDSSPLLQFGGQVRQRYLYTDDAQQTEAHLEIREDGTVGGAADQSPESLLQLKALKPGVIQILGVKTSRFLCQRPDGALYGSLHFDPEACSFRELLLEDGYNVYQSEAHGLPLHLPGNKSPHRDPAPRGPAR**FLPMLPMVPEEPEDLRGHLESDMFSSPLETDSMDPFGLVTGLEAVRSPSFEK

17 (hFGF21): **HPIPDSSPLLQFGGQVRQRYLYTDDAQQTEAHLEIREDGTVGGAADQSPESLLQLKALKPGVIQILGVKTSRFLCQRPDGALYGSLHFDPEACSFRELLLEDGYNVYQSEAHGLPLHLPGNKSPHRDPAPRGPARFLPLPGLPPALPEPPGILAPQPPDVGSSDPLSMVGPSQGRSPSYAS**

### Luciferase assay

All the cells were cultured in Dulbecco's Modified Eagle Medium (DMEM) supplemented with 10% fetal bovine serum (FBS) at 37°C under 5% CO2. Rat L6 myoblasts in a 96-well plate were transiently-transfected with expression vectors encoding Renilla luciferase (pRL-SV40, Promega), human KLB, appropriate human FGFR, GAL4-Elk-1 transcriptional activator (pFA2-Elk1, Stratagene), and firefly luciferase reporter driven GAL4 binding sites (pFR-luc, Stratagene), using FuGENE HD Transfection Reagent (Roche Applied Science). On the next day, the transfected cells were cultured for an additional 6*–*8 hours in serum free media containing 25 mg/L porcine heparin (Sigma) and FGF protein at a various concentration. The cells were then lysed with PLB reagent (Promega) and luciferase activity in each well was determined using Dual-Glo Luciferase Assay System (Promega) and EnVision Multilabel Reader (PerkinElmer). Firefly luciferase activity was normalized to the co-expressed Renilla luciferase activity, and is shown as an average and standard error of the mean of the three replicas.

### Anchorage independent cell proliferation assay

A 96-well-plate was filled with 50 µL/well of 0.5% molten agarose in growth media. After the base agarose had solidified, about 670 HepG2 cells suspended in 50 µL top molten agarose solution (0.35% agarose in growth media) were added to the base agar in each well, and allowed to solidify. Following solidification, 20 µL of growth medium containing an appropriate amount of FGF protein was added to each well on designated day 0. On each of the subsequent days 2, 4, 6 and 8, a further 20 µL of growth medium with an appropriate amount of FGF protein was added to each well. A subset of the sample wells was also treated with protein synthesis inhibitor Geneticin (Invitrogen) to provide a background fluorescence signal. On day 9, 10 µL AlamarBlue reagent (Invitrogen) was added to each sample well and the plate was further incubated for 5 hrs. The resulting fluorescent intensity was measured using EnVision Multilabel Reader (PerkinElmer) and used as an indication of the total metabolic activity in each well. Each condition was tested in quintuplicate.

### FGFR/ligand binding assay

FGFR-binding activity of FGF19 and FGF19v were measured as described in [25] using biotinylated anti-FGF19 antibody (BAF969, R&D systems) in the presence of 2 µg/mL heparin. Control ELISA experiments were performed using anti-FGF19 antibody (AF969, R&D systems) and biotinylated anti-FGF19 antibody (BAF969, R&D systems) to confirm that the antibody reacts to FGF19 and FGF19v in an indistinguishable manner.

### Mouse Studies

Mice were maintained in a pathogen-free animal facility at 21°C under standard 12 hr light/12 hr dark cycle with access to chow (a standard rodent chow (Labdiet 5010, 12.7% calories from fat) or a high fat, high carbohydrate diet (Harlan Teklad TD.03584, 58.4% calories from fat) and water ad libitum. Male mice were used for all of the experiments described. FGFR4 KO mice in C57BL/6 background were previously described [20], [29], [35]. C57BL/6 mice, ob/ob mice in C57BL/6 background and FVB/NJ mice were purchased from Jackson Laboratory. For continuous infusion of FGF protein, an osmotic pump (Alzet 2001) was subcutaneously implanted. For glucose tolerance test, glucose levels were measured using One Touch Ultra glucometer. BrdU staining was carried out as described [15] and BrdU positive hepatocytes were counted by using the Ariol automated image analysis system.

### Serum analysis

Total cholesterol, triglyceride, β-hydroxybutylate (BHB), lactate (Thermo DMA) and nonesterified fatty acid (Roche) were determined by using enzymatic reactions. Serum insulin levels were determined by ELISA (Crystal Chem). BA composition was determined by liquid chromatography-mass spectrometry analysis as previously described [36].

### Gene Expression Analysis

Tissue RNAs were isolated by using QIAzol reagent (Qiagen). cDNA was synthesize with the Quantitect Reverse Transcription Kit (Qiagen). For real time qPCR, samples were run in triplicate in the ABI Prism 7900HT (Applied Biosystems) by using SYBR green universal mix (Invitrogen) or by Taqman universal mix (Roche) and normalized by levels of 36B4. Pre-designed Quantitect primers for GK, SHP, Cyp8b1, IGFBP2, and AFP were obtained from Qiagen and all other primers were designed using primer express software (Applied Biosystems). Sequences of in-house designed primers will be provided upon request.

### Statistical Analyses

Unpaired student's t-test (two-tailed) was used for statistical analyses to compare treatment groups using Prism 5 software (Graphpad) or Excel (Microsoft). A *p*-value <0.05 was considered statistically significant. Values were presented as means+/− standard error of the mean.

## Supporting Information

Figure S1 **FGF21 and 19 activates FGFR2c and FGFR3c in the presence of KLB.** GAL-Elk1 luciferase assay in L6 cells. L6 cells were cotransfected with expression vectors for KLB and the indicated FGFR together with GAL-Elk1, SV40-Renilla Luciferase, and Gal-responsive luciferase reporter. Transfected cells were incubated with media containing increasing concentrations of FGF19 (○) or FGF21(▴) for 6 hours before luciferase assays. Transcriptional activation was assessed by the relative luciferase activity normalized by Renilla luciferase activity and expressed as relative luciferase unit (RLU).
(TIF)

Figure S2 ***In vitro*** **activity of FGF21, FGF19 and chimeric constructs.** GAL-Elk1 luciferase assay in rat L6 cells. L6 cells were cotransfected with expression vectors for KLB and the indicated FGFR together with GAL-Elk1, SV40-renilla Luciferase, and Gal-responsive firefly luciferase reporter. Transfected L6 cells were incubated for 6 hours before luciferase assays with conditioned medium from 293 cells transiently transfected with each FGF construct indicated at the bottom. The number below each group corresponds to the number of the construct as indicated in Fig. 2B. Transcriptional activation was assessed by the relative firefly luciferase activity normalized by renilla luciferase activity and expressed as relative luciferase unit (RLU). The results are shown as a fold induction over control media conditioned with mock transfected cells.
(TIF)
