# Supplementary figures and images for: FGF19 Regulates Cell Proliferation, Glucose and Bile Acid Metabolism via FGFR4-Dependent and Independent Pathways
Source: PLoS One. 2011 Mar 18;6(3):e17868. doi: 10.1371/journal.pone.0017868 (PMC3060878; doi:10.1371/journal.pone.0017868)

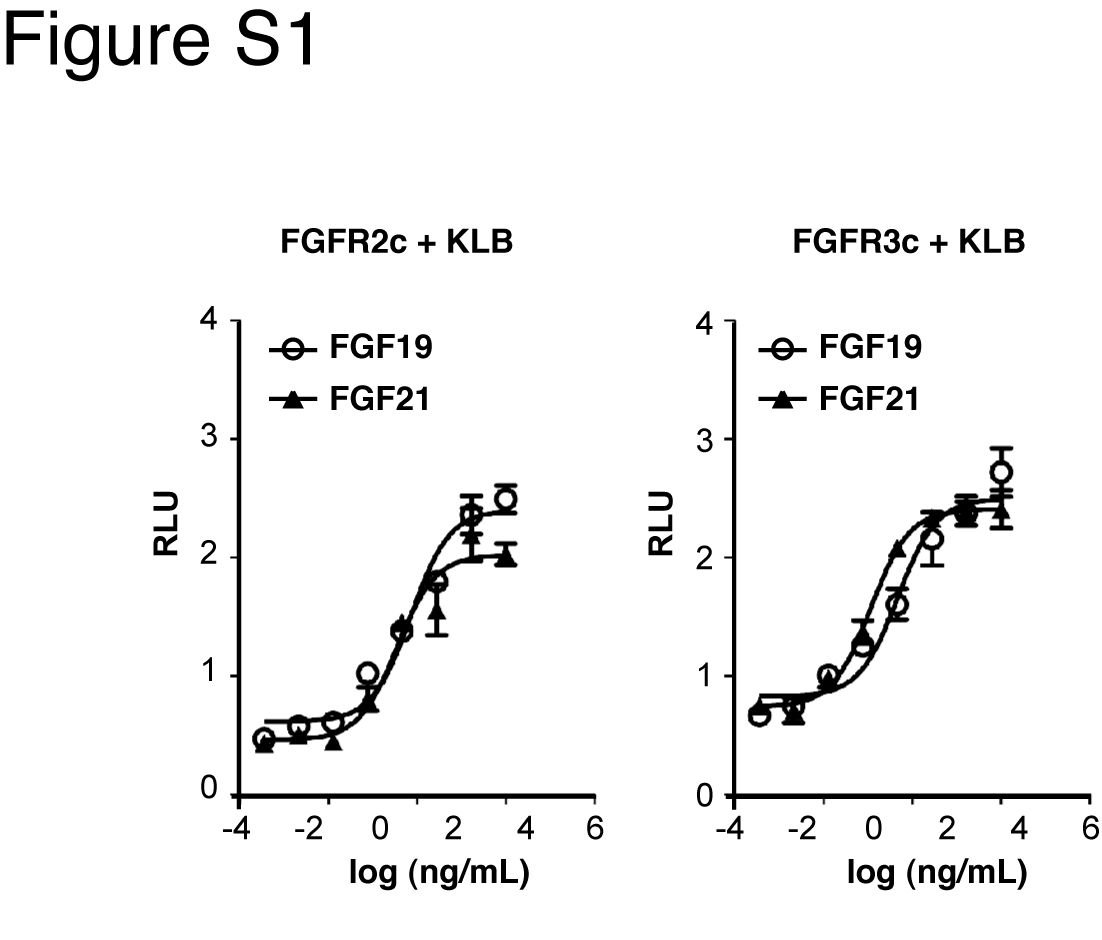

Supplement: Figure S1 — FGF21 and 19 activates FGFR2c and FGFR3c in the presence of KLB. GAL-Elk1 luciferase assay in L6 cells. L6 cells were cotransfected with expression vectors for KLB and the indicated FGFR together with GAL-Elk1, SV40-Renilla Luciferase, and Gal-responsive luciferase reporter. Transfected cells were incubated with media containing increasing concentrations of FGF19 (○) or FGF21(▴) for 6 hours before luciferase assays. Transcriptional activation was assessed by the relative luciferase activity normalized by Renilla luciferase activity and expressed as relative luciferase unit (RLU). (TIF) [file pone.0017868.s001.tif]

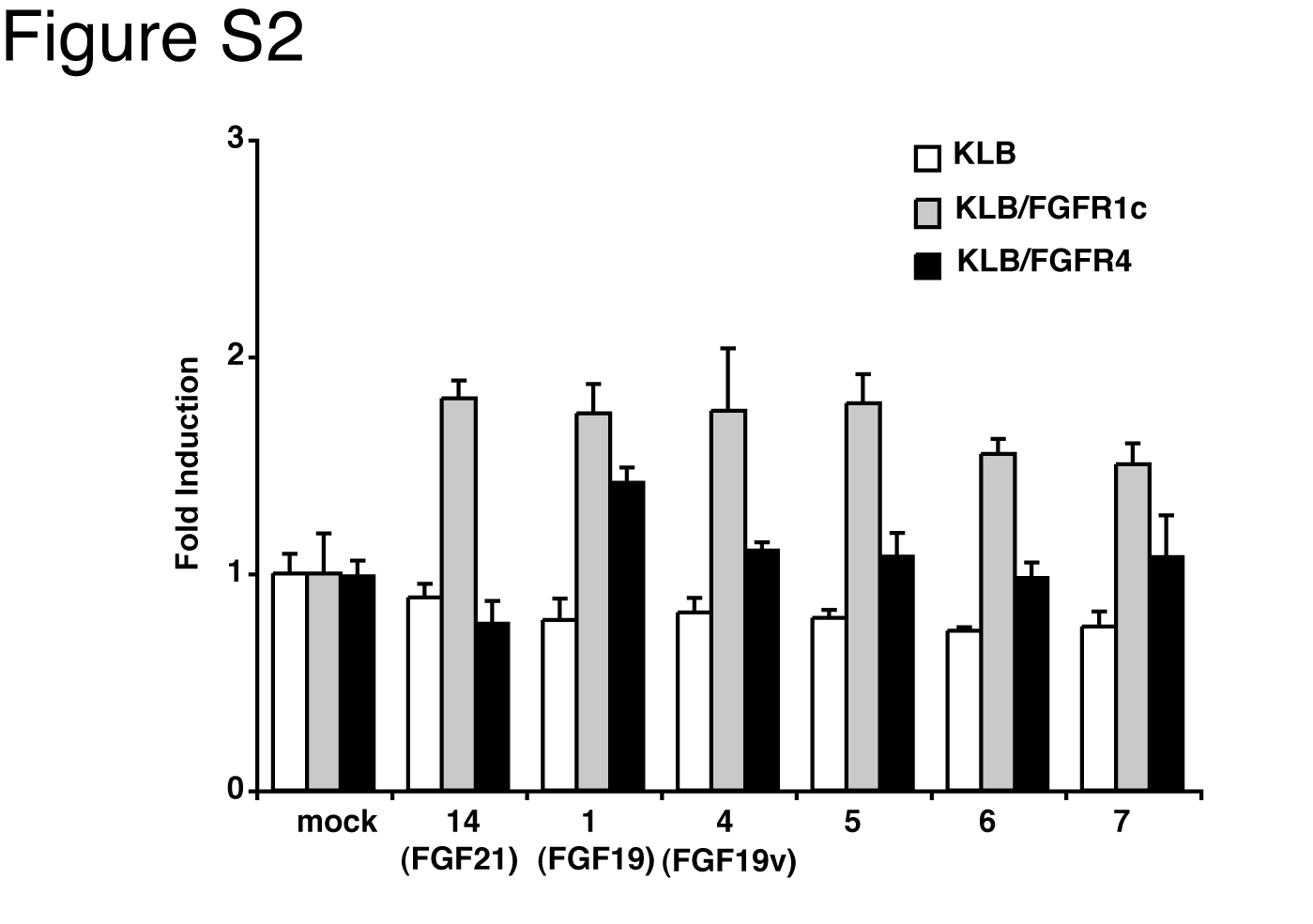

Supplement: Figure S2 — In vitro activity of FGF21, FGF19 and chimeric constructs. GAL-Elk1 luciferase assay in rat L6 cells. L6 cells were cotransfected with expression vectors for KLB and the indicated FGFR together with GAL-Elk1, SV40-renilla Luciferase, and Gal-responsive firefly luciferase reporter. Transfected L6 cells were incubated for 6 hours before luciferase assays with conditioned medium from 293 cells transiently transfected with each FGF construct indicated at the bottom. The number below each group corresponds to the number of the construct as indicated in Fig. 2B. Transcriptional activation was assessed by the relative firefly luciferase activity normalized by renilla luciferase activity and expressed as relative luciferase unit (RLU). The results are shown as a fold induction over control media conditioned with mock transfected cells. (TIF) [file pone.0017868.s002.tif]
